# Effects of COVID-19 on Mental Health and Its Relationship With Death Attitudes and Coping Styles Among Hungarian, Norwegian, and Turkish Psychology Students

**DOI:** 10.3389/fpsyg.2022.812720

**Published:** 2022-02-07

**Authors:** Kemal Oker, Melinda Reinhardt, Ágoston Schmelowszky

**Affiliations:** ^1^Doctoral School of Psychology, ELTE Eötvös Loránd University, Budapest, Hungary; ^2^Institute of Psychology, ELTE Eötvös Loránd University, Budapest, Hungary

**Keywords:** COVID-19, death attitudes, coping styles, cross-cultural comparison, mental health

## Abstract

The purpose of this study was to investigate mental effects of coronavirus disease 2019 (COVID-19) and its relationship with death attitudes and coping styles among Hungarian, Norwegian, and Turkish psychology students. A total of 388 participants from Hungary (*N* = 122, 31.4%), Norway (*N* = 96, 24.7%), and Turkey (*N* = 170, 43.8%) were recruited during the pandemic. The Depression, Anxiety and Stress Scale, the Impact of Event Scale-Revised, the Carver Brief COPE Inventory, and the Death Attitude Profile-Revised were used. The results indicated that escape acceptance might be the most maladaptive death attitude during COVID-19, as it was related to poorer mental health among the Hungarian, Norwegian, and Turkish psychology students. Self-blame, behavioral disengagement, self-distraction, and substance use coping styles were also related to poorer mental health, whereas positive-reframing (only among the Hungarian and Turkish participants) and humor (only among the Norwegian participants) were related to better mental health among our sample in the context of COVID-19. The findings implied that death attitudes and coping styles may differ in their efficacy among the Hungarian, Norwegian, and Turkish participants. These differences were discussed in detail in the discussion part. During the pandemic, practitioners might pay closer attention to patients with higher escape acceptance death attitude and patients who use dysfunctional coping styles. Additionally, patients can be encouraged to use techniques involving positive reframing and humor coping styles.

## Introduction

Coronavirus disease-2019 (COVID-19) has caused a great deal of stress in almost all countries in the world (Gormsen and Koijen, 2020; Remuzzi and Remuzzi, 2020; Vahedian-Azimi et al., 2020; Xu et al., 2020). During the early stages of the pandemic, several studies were performed to understand how COVID-19 affected the emotion and behavior of people and their antecedents based on psychological aspects (Guo et al., 2020; Li et al., 2020; Skapinakis et al., 2020; Wang et al., 2020; Zacher and Rudolph, 2020).

Evidence from previous studies (e.g., Sim et al., 2010; Guo et al., 2020; Gurvich et al., 2020; Skapinakis et al., 2020; Zacher and Rudolph, 2020) suggested that in COVID-19 and similar outbreaks, coping strategies play a crucial role, as different coping styles are related to different psychological responses that can either increase or decrease the psychological and physical wellbeing of a person (Kasi et al., 2012; Gurvich et al., 2020). Several studies (Chew et al., 2020; Fu et al., 2020; Skapinakis et al., 2020) showed that positive/active, approach/problem-focused coping strategies are more effective than negative/passive, avoidant/emotion-focused coping strategies in terms of adaptation to and mitigation of mental effects of COVID-19. For instance, Zacher and Rudolph (2020) investigated subjective wellbeing during COVID-19 in a German population. They reported that life satisfaction was positively related to active coping and positive reframing and negatively related to planning. Regarding the finding about planning, the authors explained that high levels of insecurity related to the outbreak might have turned future planning into an unpleasant experience. In addition, they reported that positive affect was positively associated with active coping, using emotional support, and religion, and negatively associated with humor. Regarding the finding about humor, the authors concluded that during the outbreak, using humor can be a less effective coping strategy and might rather constitute gallows humor. Lastly, the authors stated that negative affect was positively correlated to denial, substance use, and self-blame, and negatively correlated to using emotional support. Another research was conducted by Gurvich et al. (2020) regarding coping styles and mental health during COVID-19. The outcomes of the study showed that positive reframing, acceptance, and humor were related to better mental health, whereas self-blame, venting, behavioral disengagement, and self-distraction were associated with poorer mental health.

Death anxiety is also a very important topic when investigating the psychological impact of COVID-19 (Menzies and Menzies, 2020; Pradhan et al., 2020; Pyszczynski et al., 2020). COVID-19 has resulted in deaths of more than 1,000,000 people worldwide at the time of writing (World Health Organization, 2020). Everyday people are hearing about thousands of deaths caused by the pandemic, and this has an influence on the wellbeing of people and creates a constant fear of death in many people’s lives (Pradhan et al., 2020). Pyszczynski et al. (2020) argued that irrespective of whether an individual consciously believes that COVID-19 is a major threat to life or just a minor trouble, fear of death plays a crucial role in guiding one’s attitudes and behavior regarding the virus. Pradhan et al. (2020) also investigated the relationship of death anxiety with neuroticism and perceived stress in the context of COVID-19. The authors found that death anxiety was positively correlated with both neuroticism and perceived stress (Pradhan et al., 2020). There are also several studies (Pollak, 1980; Abdel-Khalek, 1997; Abdel-Khalek and Tomás-Sábado, 2005; Iverach et al., 2014) that demonstrated the positive relationship of death anxiety with depression, anxiety, post-traumatic stress disorder (PTSD), and stress. Moreover, death anxiety has been proposed as a transdiagnostic construct (Iverach et al., 2014), meaning that death anxiety can play a significant role in the development and severity of symptoms of several diseases, such as depression, anxiety, eating disorders, and PTSD. In addition, Yalom (1980) stated that death anxiety is a primary fear that underpins a set of mental disorders, including panic disorder, anxiety, and depression. Furthermore, previous findings have shown that attitudes toward death do not necessarily include only fear and anxiety. Thus, studies revealed that death anxiety is a multidimensional concept (Collett and Lester, 1969; Ray and Najman, 1974; Hoelter, 1979; Florian and Kravetz, 1983). In fact, Wong et al. (1994) developed a multidimensional death attitude scale (Death Attitudes Profile- Revised; DAP-R), which includes five different death attitudes: (a) fear of death (experiencing feelings of fear at a conscious level that are triggered when faced with issues related to death), (b) death avoidance (avoiding thinking or talking about death in order to decrease death anxiety), (c) approach acceptance (believing in a happy afterlife), (d) escape acceptance (believing that death is an escape from a painful life), and (e) neutral acceptance (perceiving death as a natural part of life). Studies (Gesser et al., 1988; Wong et al., 1994) showed that neutral acceptance and approach acceptance are related to better psychological wellbeing, while fear of death, death avoidance, and escape acceptance are related to poorer psychological wellbeing. Due to the fact that one’s knowledge and attitudes toward death influence the way one copes with diseases (Ho and Shiu, 1995; Nozari and Dousti, 2013; Wittkowski, 2015), investigating these five different death attitudes in the context of COVID-19 would be worthwhile in developing adaptive psychological strategies, techniques, and interventions.

The current pandemic is unique in terms of number of countries affected (Gurvich et al., 2020). In fact, 220 countries, areas, and territories have been diagnosed with cases (World Health Organization, 2020). Previous findings have suggested that for future studies, it is important to identify people prone to psychological disorders related to COVID-19 from different cultures, communities, and countries in order to deepen our understanding of psychological aspects of COVID-19 and eventually to develop adaptive psychological interventions (Salari et al., 2020; Zacher and Rudolph, 2020). Furthermore, studies have emphasized the need for follow-up studies (Xiong et al., 2020) to investigate the later stages of the pandemic in terms of its effect on mental health and in order to assess its long-term effects.

### Present Research

This study aims to detect the mental effects of COVID-19 and its relationship with death attitudes and coping styles in different countries by examining a sample composed of Hungarian, Norwegian, and Turkish psychology students. Studies on COVID-19 showed that depression, anxiety, stress, and PTSD are among the leading psychological problems in the context of the pandemic (Gurvich et al., 2020; Xiong et al., 2020). Moreover, student status is found to be associated with greater psychological impact of the COVID-19 outbreak and higher levels of stress, anxiety, and depression (Wang et al., 2020). Lee (2020) also reported that higher education is related to higher coronavirus anxiety. Lee (2020) stated that additional research about this population is needed. Therefore, we decided to conduct our research among university students and examined their depression, anxiety, stress, and PTSD levels. Based on our literature review, this will be the first study to investigate the mental effects of COVID-19 and its relationship with death attitudes and coping styles among three different countries (Hungary, Norway, and Turkey).

These countries were selected for a number of reasons. First, death attitudes may change from culture to culture (Lehto and Stein, 2009; Gire, 2014), and this difference can play a critical role in buffering the anxiety, depression, and stress related to COVID-19 (Jovančević and Milićević, 2020). In addition, before COVID-19, in 2015, we had run a research where we compared Turkish and Norwegian psychology students with respect to their death anxiety and different death attitudes and the relationship of these variables with depressive and anxiety symptoms (Oker et al., 2019, 2020). Thus, we considered it worthwhile to examine the Norwegian and Turkish university student population again with the same variables during COVID-19. In addition, according to the cultural dimensions of Hofstede et al. (2010), there are both similarities and differences among Hungary, Norway, and Turkey: Power distance (Norway and Hungary = low, Turkey = high), individualism (Hungary and Norway = individualistic, Turkey = collectivistic), masculinity (Norway = low, Turkey = middle, and Hungary = high), uncertainty avoidance (Hungary and Turkey = high, Norway = middle), long-term orientation (Hungary = high, Norway = low, and Turkey = middle), and indulgence (Hungary = low, Norway and Turkey = middle) (Hofstede Insights, 2018). Thus, it can be worthwhile to examine these three distinctive countries during the pandemic (Jovančević and Milićević, 2020). We, therefore, suggest that this study will contribute to the literature in terms of providing deeper insight to our understanding of psychological aspects of COVID-19, and eventually will help to develop culture-specific adaptive psychological interventions. Additionally, to the best of our knowledge, this study was unique in terms of examining different death attitudes related to COVID-19 among the three countries.

As there is no previous research that compares these countries in the context of the pandemic related to death attitudes and coping strategies and because of the novelty of the virus, no specific hypotheses were drawn in this study. Therefore, this study is exploratory research. The main aim of this study is to check how these three distinctive countries may differ in terms of the effects of COVID-19 on mental health and its relationship with death attitudes and coping styles among psychology students. More specifically, we are interested in examining the relationship of approach coping, avoidant coping, humor, and religion coping styles with depression, anxiety, stress, and PTSD symptoms among the Hungarian, Norwegian, and Turkish participants. In addition, we are interested in exploring the relationship between the five different death attitudes (fear of death, death avoidance, neutral acceptance, approach acceptance, and escape acceptance) and depression, anxiety, stress, and PTSD symptoms among the three countries. Lastly, we are interested in investigating the relationship of the approach coping, avoidant coping, humor, and religion coping styles with the five different death attitudes among the three countries.

## Method

### Participants

This study included a total of 388 (female = 328, 84.5% and male = 60, 15.5%) participants from Hungary (*N* = 122, 31.4%), Norway (*N* = 96, 24.7%), and Turkey (*N* = 170, 43.8%). The participants were students of psychology from different universities in Hungary, Norway, and Turkey. Participant age ranged from 18 to 60 years (*M* = 24.2, SD = 6). Inclusion criteria were based on participant age (equal to or over 18 years old), whether they were studying psychology, and whether they were able to read and understand English. Convenience sampling method was used. There were several reasons for choosing psychology students. First, we had greater access to psychology students, and they were more responsive and more willing to volunteer to participate in the research. Second, our aim was to keep the sample homogeneous in terms of education and knowledge. Table 1 presents the details of sample characteristics.

**TABLE 1:** Characteristics of the sample.

Characteristic	Hungary (*N* = 122; 31.4%)	Norway (*N* = 96; 24.7%)	Turkey (*N* = 170; 43.8%)
**Gender** Female Male	101 (82.8) 21 (17.2)	75 (78.1) 21 (21.9)	152 (89.4) 18 (10.6)
**Economic Status** Below Average Above average Missing	8 (6.6) 89 (73.0) 25 (20.5) 0	13 (13.5) 66 (68.8) 17 (17.7) 0	5 (2.9) 128 (75.3) 36 (21.2) 1 (0.6)
**Education** BA MA PhD	70 (57.4) 43 (35.2) 9 (7.4)	29 (30.2) 55 (57.3) 12 (12.5)	101 (59.4) 40 (23.5) 29 (17.1)
**Marital Status** Married Divorced Single With a partner Other **Relationship with the COVID-19** Healthy Suspicious case Diagnosed case Relatives or friends of suspicious case Relatives or friends of diagnosed case Other **History of chronic illness** No Yes Missing **Current physical health condition** Very poor Poor Average Good Very good	5 (4.1) 1 (0.8) 60 (49.2) 55 (45.1) 1 (0.8) 105 (86.1) 5 (4.1) 2 (1.6) 12 (9.8) 16 (13.1) 1 (0.8) 111 (91.0) 10 (8.2) 1 (0.8) 1 (0.8) 3 (2.5) 41 (33.6) 45 (36.9) 32 (26.2)	8 (8.3) 1 (1.0) 47 (49.0) 39 (40.6) 1 (1.0) 86 (89.6) 3 (3.1) 3 (3.1) 1 (1.0) 7 (7.3) 0 84 (87.5) 12 (12.5) 0 0 5 (5.2) 30 (31.3) 40 (41.7) 21 (21.9)	15 (8.8) 4 (2.4) 99 (58.2) 51 (30.0) 1 (0.6) 150 (88.2) 4 (2.4) 2 (1.2) 5 (2.9) 19 (11.2) 1 (0.6) 137 (80.6) 33 (19.4) 0 0 8 (4.7) 40 (23.5) 80 (47.1) 42 (24.7)

*Data are presented as N (%).*

### Measures

The research was based on five scales, including the demographic scale which was comprising questions about the participants’ nationality, gender, age, education, economic status, marital status, current residential location, relationship with COVID-19, history of chronic illness and current physical health condition. All the respondents were given the English version of the scales. Participants were fluent in English, as the majority of participants’ language of education in their university was English.

Mental health status was measured using the Depression, Anxiety and Stress Scales (DASS-21) (Lovibond and Lovibond, 1995). The scale is a set of three self-report scales designed to assess the emotional states of depression, anxiety, and stress. Items 3, 5, 10, 13, 16, 17, and 21 formed the depression subscale (example item: “I couldn’t seem to experience any positive feeling at all”). The total depression subscale score was divided into normal (0–9), mild depression (10–12), moderate depression (13–20), severe depression (21–27), and extremely severe depression (28–42). Items 2, 4, 7, 9, 15, 19, and 20 formed the anxiety subscale (example item: “I felt I was close to panic”). The total anxiety subscale score was divided into normal (0–6), mild anxiety (7–9), moderate anxiety (10–14), severe anxiety (15–19), and extremely severe anxiety (20–42). Items 1, 6, 8, 11, 12, 14, and 18 formed the stress subscale (example item: “I found myself getting agitated”). The total stress subscale score was divided into normal (0–10), mild stress (11–18), moderate stress (19–26), severe stress (27–34), and extremely severe stress (35–42). In this research, Cronbach’s alpha for the depression subscale was 0.91 for Hungary, 0.92 for Norway, and 0.9 for Turkey. For the anxiety subscale, it was 0.81 for Hungary, 0.76 for Norway, and 0.75 for Turkey. Lastly, for the stress subscales, it was 0.82 for Hungary and 0.87 for Norway and Turkey.

The psychological impact of COVID-19 was measured using the Impact of Event Scale-Revised (IES-R; Weiss and Marmar, 1997). The IES-R is a self-administered 22-item questionnaire (example item: “any reminder brought back feelings about it”). In this study, the participants were asked to reply to questions with respect to COVID-19. The total IES-R score was divided into 0–23 (normal), 24–32 (mild psychological impact), 33–36 (moderate psychological impact), and >37 (severe psychological impact) (Creamer et al., 2003). In this study, Cronbach’s alpha for this test was 0.90 for Hungary, 0.95 for Norway, and 0.93 for Turkey.

Coping styles were measured with the Brief COPE Inventory (Carver, 1997). The Brief COPE Inventory can identify 14 coping styles with 28 questions and 2 items per type. The scale includes two main coping style dimensions: approach and avoidant coping styles. Avoidant coping is characterized by the subscales of denial (α = 0.68 for Hungary; α = 0.8 for Norway; α = 0.7 for Turkey), an example item of the subscale is, “I’ve been refusing to believe that it has happened”; substance use (α = 0.95 for Hungary; α = 0.92 for Norway; α = 0.92 for Turkey), an example item of the subscale is, “I’ve been using alcohol or other drugs to make myself feel better”; venting (α = 0.51 for Hungary; α = 0.5 for Norway; α = 0.21 for Turkey), an example item of the subscale is: “I’ve been expressing my negative feelings”; behavioral disengagement (α = 0.74 for Hungary; α = 0.65 for Norway; α = 0.68 for Turkey), an example item of the subscale is: “I’ve been giving up the attempt to cope”, self-distraction (α = 0.63 for Hungary; α = 0.71 for Norway; α = 0.61 for Turkey), an example item of the subscale is, “I’ve been turning to work or other activities to take my mind off things”; and self-blame (α = 0.55 for Hungary; α = 0.75 for Norway; α = 0.52 for Turkey), an example item of the subscale is, “I’ve been blaming myself for things that happened”. Approach coping is characterized by the subscales of active coping (α = 0.68 for Hungary; α = 0.72 for Norway; α = 0.64 for Turkey), an example item of the subscale is, “I’ve been taking action to try to make the situation better”; positive reframing (α = 0.81 for Hungary; α = 0.75 for Norway; α = 0.8 for Turkey), an example item of the subscale is, “I’ve been looking for something good in what is happening”; planning (α = 0.81 for Hungary; α = 0.75 for Norway; α = 0.5 for Turkey), an example item of the subscale is, “I’ve been thinking hard about what steps to take”; acceptance (α = 0.58 for Hungary; α = 0.83 for Norway; α = 0.6 for Turkey), an example item of the subscale is, “I’ve been accepting the reality of the fact that it has happened”; use of emotional support (α = 0.8 for Hungary; α = 0.89 for Norway; α = 0.66 for Turkey), an example item of the subscale is, “I’ve been getting emotional support from others”; and use of instrumental support (α = 0.82 for Hungary; α = 0.89 for Norway; α = 0.77 for Turkey), an example item of the subscale is, “I’ve been getting help and advice from other people”. According to the scale, humor (α = 0.9 for Hungary; α = 0.9 for Norway; α = 0.85 for Turkey) and religion (α = 0.81 for Hungary; α = 0.72 for Norway; α = 0.78 for Turkey) coping styles do not belong to neither approach nor avoidant coping. An example item for the humor subscale is, “I’ve been making jokes about it” and for the religion is, “I’ve been trying to find comfort in my religion or spiritual beliefs”. In this study, the respondents were asked to read the statements and indicate how much they have been using each coping style to cope with COVID-19-related stress symptoms. With short scales (e.g., scales including less than 5 items), it is usual to observe low Cronbach’s alpha (Briggs and Cheek, 1986). Because in the BRIEF COPE measurement each subscale includes only 2 items, some subscales showed low Cronbach’s alpha in this study. However, since the subscales had only 2 items, we can consider an alpha score from 0.5 to 0.7 as showing moderate reliability (Hinton et al., 2014, p. 359). However, for the Turkish participants, the venting coping style subscale was lower than 0.5. Therefore, we excluded this subscale from further analysis.

Death attitudes were measured by the Death Attitude Profile-Revised scale (DAP-R; Wong et al., 1994). It comprises 32 items and five death attitudes: (a) fear of death (α = 0.85 for Hungary and Norway; α = 0.79 for Turkey), and an example item of the subscale is, “I have an intense fear of death”; (b) death avoidance (α = 0.91 for Hungary; α = 0.9 for Norway; and α = 0.81 for Turkey), an example item of the subscale is, “I avoid death thoughts at all costs”; (c) approach acceptance (α = 0.9 for Hungary; α = 0.93 for Norway; and α = 0.91 for Turkey), an example item of the subscale is, “Death is an entrance to a place of ultimate satisfaction”; (d) escape acceptance (α = 0.84 for Hungary; α = 0.89 for Norway; and α = 0.85 for Turkey), an example item of the subscale is, “Death will bring an end to all my troubles”, and (e) neutral acceptance (α = 0.7 for Hungary; α = 0.69 for Norway; and α = 0.69 for Turkey), an example item of the subscale is “Death should be viewed as a natural, undeniable, and unavoidable event”.

### Procedure

Several psychology lecturers in different universities in Hungary, Norway, and Turkey were contacted to share our survey link with their students. The survey was administered online. Data were collected between July 2 and November 20, 2020. At the beginning of the survey, the participants were given detailed written information about the research, and informed consent was obtained voluntarily from all the participants. Ethical permission was obtained from the Institution of Review Board of ELTE Eötvös Loránd University (reference number: 2020/166).

### Data Analysis

The Statistical Package for the Social Sciences (SPSS) 20.0 software was used to perform our analyses. Accordingly, descriptive statistics was used to summarize the characteristics of the sample all the participants. In addition, four separate hierarchical multiple linear regressions were applied to predict depressive, anxiety, stress, and PTSD symptoms from the five death attitudes and from the thirteen different coping styles for the three countries, after controlling for age and gender. The regression analyses were carried out in three steps: In the first step, age and gender control variables were entered as predictors. In the second step, the five different death attitudes were added. In the third step, the thirteen different coping styles were entered in the regression model. For the anxiety analysis part, case-wise diagnostic detected two outliers from Turkey (case numbers 273 and 313). These cases were filtered out from the analysis, and then the analysis was re-run. Lastly, a set of Pearson correlation tests were also run to assess the relationship between the five death attitudes and the thirteen coping styles.

## Results

The findings indicate that among the Hungarians, none of the control variables were significantly correlated with stress in any of the steps. Moreover, the fear of death and escape acceptance death attitudes were significantly positively related to stress in step 2, whereas only the escape acceptance death attitude remained significantly related to stress in step 3. In addition, the positive reframing and self-blame coping styles were significantly related to stress. Positive reframing was negatively associated, and self-blame was positively associated with stress. For the Norwegian participants, none of the control variables were significantly correlated with stress in steps 1 and 2. However, age was significantly and positively correlated with stress, after the coping styles were entered into the regression in step 3. Furthermore, the fear of death and escape acceptance death attitudes were significantly positively related to stress in step 2, while none of the death attitudes remained significantly correlated with stress in step 3. In addition, the emotional support and self-blame coping styles were significantly related with stress. The analysis showed that the both emotional support and self-blame coping styles were positively associated with stress among the Norwegians. For the Turkish participants, age was significantly and negatively correlated with stress in all the steps. In addition, fear of death and escape acceptance significantly positively predicted stress in step 2. Based on the results, both fear of death and escape acceptance death attitudes remained significantly associated with stress in step 3. Moreover, the behavioral disengagement, positive reframing, and self-blame coping styles were also significantly correlated with stress among the Turkish participants. The analysis showed that the self-blame and behavioral disengagement coping styles were positively related to and positive reframing was negatively related to stress. Table 2 presents the details of the hierarchical multiple regression analysis for stress.

**TABLE 2:** Summary of the hierarchical multiple regression analysis for the prediction of stress in the three countries.

	Hungary			Norway			Turkey		
	Step 1	Step 2	Step 3	Step 1	Step 2	Step 3	Step 1	Step 2	Step 3
***Control variables***									
Age	0.01	–0.02	–0.10	–0.07	0.04	0.19*	–0.31**	–0.25**	–0.14*
Gender	0.15	0.15	0.14	0.13	0.15	0.04	0.08	0.08	0.12
***Independent variables***									
Fear of death		0.26*	0.17		0.30*	0.11		0.23**	0.21**
Death avoidance		0.10	–0.02		0.12	0.10		–0.06	–0.10
Neutral acceptance		–0.07	–0.14		0.03	0.05		0.03	0.01
Approach Acceptance		0.03	0.00		0.02	0.01		–0.06	0.05
Escape Acceptance		0.42**	0.28**		0.21*	0.09		0.31**	0.21**
Self-distraction			0.11			0.21			0.11
Active coping			0.14			–0.02			0.02
Denial			0.01			0.00			–0.12
Substance use			0.05			0.08			0.13
Emotional support			–0.13			0.34*			0.08
Informational support			0.16			–0.21			–0.08
Behavioral disengagement			0.10			0.13			0.16*
Positive reframing			–0.32**			–0.22			–0.22**
Planning			0.18			–0.02			0.02
Humor			0.00			–0.13			–0.08
Acceptance			0.03			–0.09			0.01
Religion			0.08			0.11			0.04
Self-blame			0.30**			0.52**			0.35**
R^2^	0.02	0.20	0.49	0.02	0.18	0.58	0.11	0.25	0.50
Adjusted R^2^	0.01	0.15	0.39	0.00	0.11	0.47	0.10	0.21	0.43
R^2^ –changed	0.02	0.17	0.29	0.02	0.15	0.40	0.11	0.14	0.25
F	1.40	3.96**	4.84**	1.14	2.71*	5.20**	10.28**	7.57**	7.30**
N	122	122	122	96	96	96	170	170	170

Figures shown are standardized coefficients (i.e., beta values). *p < 0.05, **p < 0.01.

Furthermore, the findings indicate that for Hungarians, none of the control variables were significantly correlated with depression in any of the steps. In addition, only escape acceptance death attitude was significantly positively related to depression in both steps 2 and 3. The substance use and self-blame coping styles were also significantly positively related to depressive symptoms among the Hungarian respondents. For the Norwegian respondents, age was significantly and negatively correlated with depression in step 1, but it was not in steps 2 and 3. Moreover, only the escape acceptance death attitude was significantly positively related to depression in both steps 2 and 3. The behavioral disengagement and self-blame coping styles were also significantly positively related to depressive symptoms. For the Turkish sample, age was significantly and negatively correlated with depression in steps 1 and 2, but it was not in step 3. Furthermore, the fear of death and escape acceptance death attitudes were significantly and positively correlated with depressive symptoms in both steps 2 and 3. The self-distraction, behavioral disengagement, and self-blame coping styles were significantly positively related to and positive-reframing was significantly negatively related to depressive symptoms among the Turkish participants. Table 3 presents the details of the hierarchical multiple regression analysis for depression.

**TABLE 3:** Summary of the hierarchical multiple regression analysis for the prediction of depression in the three countries.

	Hungary			Norway			Turkey		
	Step 1	Step 2	Step 3	Step 1	Step 2	Step 3	Step 1	Step 2	Step 3
***Control variables***									
Age	–0.01	–0.07	–0.09	–0.24*	–0.17	–0.04	–0.31**	–0.26**	–0.11
Gender	–0.06	–0.06	–0.10	0.02	0.11	0.00	–0.02	–0.01	0.02
***Independent variables***									
Fear of death		0.17	0.04		0.17	0.06		0.20*	0.17*
Death avoidance		0.10	–0.07		0.10	0.03		–0.09	–0.12
Neutral acceptance		–0.08	–0.15		0.09	0.07		0.03	0.02
Approach acceptance		–0.09	–0.04		–0.11	–0.08		–0.11	0.04
Escape acceptance		0.46**	0.30**		0.44**	0.31**		0.35**	0.20**
Self-distraction			0.06			0.09			0.16*
Active coping			–0.06			0.05			–0.07
Denial			0.16			0.03			–0.01
Substance use			0.26**			–0.02			0.03
Emotional support			–0.06			0.08			0.05
Informational support			0.01			–0.13			–0.13
Behavioral disengagement			0.04			0.33**			0.26**
Positive reframing			–0.15			–0.20			–0.23**
Planning			0.12			–0.11			0.02
Humor			–0.02			–0.09			0.00
Acceptance			0.02			–0.01			0.02
Religion			–0.01			0.07			0.01
Self-blame			0.32**			0.43**			0.30**
R^2^	0.00	0.18	0.49	0.06	0.24	0.56	0.10	0.25	0.52
Adjusted R^2^	–0.01	0.12	0.39	0.04	0.18	0.44	0.09	0.22	0.46
R^2^ –changed	0.00	0.17	0.32	0.06	0.18	0.32	0.10	0.15	0.27
F	0.20	3.46**	4.90**	3.03	4.00**	4.74**	8.99**	7.69**	8.21**
N	122	122	122	96	96	96	170	170	170

Figures shown are standardized coefficients (i.e., beta values). *p < 0.05, **p < 0.01.

For the Hungarian participants, the analysis related to anxiety revealed that gender predicted anxiety significantly in all the steps, and that age was significantly and negatively related to anxiety only in step 3. In addition, only the escape acceptance death attitude was significantly positively related to depression in both steps 2 and 3. Substance use, self-blame coping, and active coping styles were significantly positively related to, and emotional support and positive reframing were negatively related to anxiety. For the Norwegian participants, none of the control variables were significantly correlated with anxiety in any of the steps. Similarly, none of the death attitudes were significantly correlated with anxiety in both steps 2 and 3, whereas the self- blame and religion coping styles were significantly positively related to and humor coping style was significantly negatively related to anxiety. For the Turkish sample, age was significantly and negatively correlated with anxiety in all the steps. Moreover, the fear of death and escape acceptance death attitudes were significantly and positively correlated with anxiety in step 2. However, in the third step, fear of death was not significantly related to anxiety; instead, the neutral acceptance and escape acceptance death attitudes were significantly and positively correlated with anxiety in step 3. Moreover, only the behavioral disengagement coping style was significantly positively correlated with anxiety. Table 4 presents the details of the hierarchical multiple regression analysis for anxiety.

**TABLE 4:** Summary of the hierarchical multiple regression analysis for the prediction of anxiety in the three countries.

	Hungary			Norway			Turkey		
	Step 1	Step 2	Step 3	Step 1	Step 2	Step 3	Step 1	Step 2	Step 3
***Control variables***									
Age	–0.15	–0.16	–0.28**	–0.17	–0.10	–0.03	–0.28**	–0.23**	–0.18*
Gender	0.21*	0.19*	0.18*	0.13	0.12	–0.01	0.06	0.06	0.11
***Independent variables***									
Fear of death		0.16	0.06		0.15	0.08		0.21*	0.16
Death avoidance		0.14	0.00		0.14	0.03		–0.12	–0.13
Neutral acceptance		0.00	–0.08		–0.03	–0.03		0.15	0.16*
Approach acceptance		0.04	0.02		0.08	0.03		–0.02	0.09
Escape acceptance		0.42**	0.28**		0.13	–0.06		0.30**	0.22**
Self-distraction			–0.06			0.03			–0.04
Active coping			0.27**			0.01			0.02
Denial			–0.06			0.20			0.10
Substance use			0.20*			0.17			0.05
Emotional support			–0.23*			0.04			0.03
Informational support			0.09			–0.04			–0.04
Behavioral disengagement			0.14			0.20			0.32**
Positive reframing			–0.22*			–0.12			–0.04
Planning			0.12			0.12			0.10
Humor			0.00			–0.21*			–0.04
Acceptance			0.00			–0.01			0.02
Religion			0.10			0.22*			–0.02
Self-blame			0.37**			0.36**			0.07
R^2^	0.06	0.21	0.54	0.05	0.15	0.55	0.08	0.22	0.39
Adjusted R^2^	0.04	0.16	0.45	0.03	0.08	0.43	0.07	0.18	0.31
R^2^ –changed	0.06	0.15	0.33	0.05	0.09	0.41	0.08	0.13	0.18
F	3.78*	4.35**	5.96**	2.48	2.14*	4.61**	7.62**	6.31**	4.73**
N	122	122	122	96	96	96	170	170	168

Figures shown are standardized coefficients (i.e., beta values). *p < 0.05, **p < 0.01.

The analysis related to PTSD symptoms demonstrated that for the Hungarian participants, gender predicted PTSD symptoms significantly in steps 1 and 2, but that the significant relationship disappeared in step 3. Contrary to gender, age was not significantly related to anxiety in steps 1 and 2; however, it was significantly and negatively related to PTSD symptoms in step 3. In addition, fear of death, death avoidance, and escape acceptance were significantly positively related to PTSD symptoms in step 2. However, fear of death and death avoidance were not significantly correlated with PTSD symptoms in step 3; instead, approach acceptance was significantly negatively related to and escape acceptance was positively related to PTSD symptoms in step 3. Moreover, self-blame, substance use, religion, and active coping were significantly positively related to PTSD symptoms. For the Norwegian respondents, age was significantly and negatively correlated with PTSD symptoms in steps 1 and s 2, but it was not in step 3. In addition, only the escape acceptance death attitude was significantly positively related to PTSD symptoms in step 2. However, none of the death attitudes were significantly correlated with PTSD symptoms in step 3. On the other hand, the self-distraction, behavioral disengagement and self-blame coping styles were significantly positively related and humor was negatively related to PTSD symptoms. For the Turkish participants, age was significantly and negatively correlated with PTSD symptoms in all the steps. In addition, the fear of death and escape acceptance death attitudes were significantly and positively correlated with PTSD symptoms in step 2, whereas none of the death attitudes were significantly correlated with PTSD symptoms in step 3. Lastly, the behavioral disengagement and self-blame coping styles were significantly positively related to and positive-reframing was significantly negatively related to PTSD symptoms. Table 5 presents the details of the hierarchical multiple regression analysis for PTSD.

**TABLE 5:** Summary of the hierarchical multiple regression analysis for the prediction of post-traumatic stress disorder (PTSD) symptoms in the three countries.

	Hungary			Norway			Turkey		
	Step 1	Step 2	Step 3	Step 1	Step 2	Step 3	Step 1	Step 2	Step 3
***Control variables***									
Age	–0.12	–0.10	–0.18*	–0.29**	–0.23*	–0.07	–0.28**	–0.23**	–0.14*
Gender	0.23*	0.21*	0.13	0.10	0.12	–0.03	0.00	0.00	0.05
***Independent variables***									
Fear of death		0.24*	0.13		0.09	–0.07		0.19*	0.12
Death avoidance		0.23*	0.04		0.15	0.03		0.03	–0.01
Neutral acceptance		0.11	0.05		0.06	0.02		0.05	0.06
Approach acceptance		–0.10	–0.21**		0.01	0.02		–0.12	0.12
Escape acceptance		0.36**	0.19*		0.19*	–0.04		0.26**	0.12
Self-distraction			0.14			0.26*			0.05
Active coping			0.32**			–0.12			0.11
Denial			0.05			0.09			0.03
Substance use			0.18*			0.13			0.12
Emotional support			–0.15			–0.03			0.16
Informational support			0.15			0.04			–0.04
Behavioral disengagement			0.16			0.36**			0.34**
Positive reframing			–0.08			0.01			–0.16*
Planning			–0.06			0.15			0.06
Humor			–0.01			–0.20*			–0.01
Acceptance			–0.05			0.01			–0.08
Religion			0.16*			0.01			–0.13
Self-blame			0.33**			0.25*			0.19*
R^2^	0.06	0.22	0.64	0.10	0.16	0.62	0.08	0.19	0.53
Adjusted R^2^	0.04	0.18	0.57	0.08	0.09	0.52	0.07	0.16	0.46
R^2^ –changed	0.06	0.16	0.42	0.10	0.06	0.46	0.08	0.11	0.34
F	3.70*	4.69**	9.16**	5.05**	2.37*	6.13**	6.95**	5.44**	8.34**
N	122	122	122	96	96	96	170	170	170

Figures shown are standardized coefficients (i.e., beta values). *p < 0.05, **p < 0.01.

Lastly, a set of Pearson correlation tests were run to assess the relationship between the five death attitudes and the thirteen coping styles (see Table 6). According to the analysis, the significant correlations are as follows: for the Hungarian subsample, death avoidance was positively associated with denial and behavioral disengagement and negatively correlated with the acceptance coping styles. Neutral acceptance was positively associated with humor. Approach acceptance was positively correlated with emotional support and religion. Escape acceptance was positively correlated with behavioral-disengagement. For the Norwegian subsample, fear of death was associated positively with self-distraction, emotional support, informational support, behavioral disengagement, and self-blame. Death avoidance was positively correlated with self-distraction, informational support, and self-blame, whereas it was negatively correlated with humor. Neutral acceptance was negatively correlated with emotional support and positively correlated with acceptance. Approach acceptance was positively associated with positive reframing and religion. Escape acceptance was positively associated with denial and humor. For the Turkish subsample, fear of death was associated positively with self-distraction, emotional support, and denial. Death avoidance was positively associated with self-distraction. Neutral acceptance was negatively correlated with denial and positively correlated with acceptance. Approach acceptance was negatively associated with substance use and behavioral disengagement, whereas it was positively correlated with religion and positive reframing. Escape acceptance was negatively associated with active coping, positive reframing, and acceptance, while it was positively associated with behavioral disengagement and humor.

**TABLE 6:** Correlation analysis results.

	Hungary death attitudes					Norway death attitudes					Turkey death attitudes				
Coping Styles	FD	DA	NA	AA	EA	FD	DA	NA	AA	EA	FD	DA	NA	AA	EA
Self-distraction	0.03	0.10	0.05	0.03	0.13	0.39**	0.25*	–0.07	0.16	0.13	0.15*	0.17*	0.01	0.07	0.01
Active coping	–0.09	–0.09	0.07	0.14	–0.03	0.16	0.13	–0.01	0.17	0.03	0.02	0.09	0.02	0.03	–0.17*
Denial	0.15	0.30**	–0.08	0.03	–0.13	–0.05	0.12	–0.03	0.19	0.23*	0.22**	0.13	–0.33**	–0.04	0.07
Substance use	0.15	0.17	0.03	–0.04	0.09	0.00	–0.08	–0.07	–0.04	0.13	0.15	0.14	–0.02	–0.22**	0.04
Emotional support	–0.05	–0.08	0.06	0.22*	0.05	0.26**	0.09	–0.20*	0.10	0.02	0.18*	0.04	–0.05	0.05	0.01
Informational support	0.04	0.01	0.03	0.15	0.02	0.25*	0.22*	–0.19	0.16	0.03	0.11	0.03	–0.03	0.01	–0.01
Behavioral disengagement	0.08	0.23**	–0.08	0.10	0.21*	0.21*	0.14	–0.07	0.03	0.19	0.07	0.07	0.00	–0.16*	0.20*
Positive reframing	–0.08	–0.05	0.12	0.06	–0.01	0.10	0.02	0.10	0.28**	0.15	0.03	–0.02	0.08	0.30**	–0.16*
Planning	–0.07	–0.08	0.17	–0.05	0.05	0.15	0.14	0.02	0.14	0.17	0.12	0.03	0.08	0.06	0.02
Humor	–0.10	–0.06	0.31**	–0.01	0.14	–0.11	–26*	0.14	0.15	0.23*	0.13	–0.06	0.08	–0.02	0.16*
Acceptance	–0.07	–0.22*	0.10	–0.09	0.03	–0.08	–0.09	0.29**	–0.11	0.02	–0.01	0.01	0.25**	0.12	–0.16*
Religion	–0.04	–0.01	0.01	0.40**	0.06	0.01	–0.16	–0.04	0.42**	0.19	0.01	–0.10	–0.11	0.58**	0.03
Self-blame	0.15	0.12	0.03	–0.03	0.14	0.21*	0.24*	0.03	0.10	0.11	0.10	0.05	–0.01	–0.03	0.15

*Correlation is significant at the 0.05 level (2-tailed), **correlation is significant at the.01 level (2-tailed); FD, fear of death; DA, death avoidance; NA, neutral acceptance; AA, approach acceptance; EA, escape acceptance.

## Discussion

The results of this study suggest that escape acceptance might be the most maladaptive death attitude among the Hungarian, Norwegian, and Turkish participants in the context of COVID-19, since it was the only death attitude that was found to be significantly correlated with poorer mental health among the three countries alike during COVID-19. This finding was consistent with our previous research (Oker et al., 2020), which was conducted before the pandemic, in which we posited that escape acceptance may be the most maladaptive death attitude among Norwegian and Turkish university students. As explained by Wong et al. (1994), individuals with escape acceptance death attitude are usually incompetent to cope effectively with the pain and problems of existence. Consistent with this statement, the analyses of this study showed that escape acceptance was correlated with some dysfunctional coping styles, such as behavioral disengagement. Thus, having both escape acceptance death attitude and ineffective coping styles can be the plausible explanation for significantly lower mental health in our sample with high escape acceptance death attitude. Additionally, fear of death was significantly related to higher stress among the three countries alike in step 2. However, this relationship was not significant after the coping styles were entered into the regression in step 3 among the Hungarian and Norwegian participants. On the other hand, fear of death remained significantly and positively related to stress and depression among the Turkish individuals in step 3. One of the plausible explanations for this discrepancy between the Turkish and Hungarian individuals can be that for the Turkish people, the fear of death attitude was associated with some dysfunctional coping styles, namely, self-distraction and denial. However, for the Hungarian participants, the fear of death attitude was not correlated with any dysfunctional coping styles. Therefore, having both the fear of death attitude and some dysfunctional coping strategies among the Turkish respondents might have created this difference between the Turkish and Hungarian participants. With respect to the difference between the Turkish and Norwegian participants, distinction in socioeconomic status might be one of the possible explanations, as in several studies higher socioeconomic status was found to be related to lower levels of stress, depression, anxiety, and death anxiety (Iverach et al., 2014; Freeman et al., 2016). As the socioeconomic status of Norway is higher than that of Turkey, this might explain the differences between the Turkish and Norwegian participants in our sample.

The analyses of this study exhibited that the self-blame, behavioral disengagement, self-distraction, and substance use coping styles were related to poorer mental health during COVID-19 in our sample. We may conclude that self-blame might be the most maladaptive coping style, as it was associated with poorer mental health among the three countries alike during COVID-19. This result was consistent with previous studies (Gurvich et al., 2020). The items of self-blame in the questionnaire included the statements “I’ve been criticizing myself” and “I’ve been blaming myself for things that happened.” Therefore, criticizing and appraising oneself as responsible for the possible unfortunate outcomes of COVID-19 can be destructive for the mental health of an individual. In addition, behavioral disengagement can be particularly risky for the Turkish participants during COVID-19, as it was related to higher stress, depression, anxiety, and PTSD symptoms among them. Similarly, substance use might be particularly risky for the Hungarian participants during COVID-19, as it was related to higher depression, anxiety, and PTSD symptoms among them.

Positive reframing might be the most adaptive coping style among the Hungarian and Turkish participants, since it was related to better mental health among them. Accordingly, positive reframing was related to lower stress, depression, and PTSD symptoms among the Turkish participants. Moreover, positive reframing was related to lower stress and anxiety among the Hungarian participants. Therefore, it seems that reappraising the current circumstances by placing them in a positive frame was useful for the Turkish and Hungarian participants during COVID-19. This finding was consistent with previous studies (Gurvich et al., 2020; Zacher and Rudolph, 2020). Positive reframing was also found as one of the most adaptive coping strategies and a buffering factor against distress among frontline healthcare workers during the pandemic (Fino et al., 2021a). Therefore, positive reframing might be an adaptive strategy during COVID-19 for a variety of circumstances and people including frontline healthcare workers who are exposed to intense distress during the pandemic. For the Norwegians, however, humor can be the most successful coping style in the context of COVID-19, as it was related to lower anxiety and PTSD symptoms among the Norwegian respondents. This finding was consistent with the results of Gurvich et al. (2020), who found that humor was associated with better mental health among the Australian population. However, Zacher and Rudolph (2020) found that humor was negatively related to positive affect among German participants. These conflicting results suggest that the function of the humor coping style may change from culture to culture. Additionally, the style of humor (e.g., affiliative, aggressive, self-enhancing, or self-defeating) might influence the effectiveness of this coping style. Future studies may investigate this in more detail.

The findings yielded some other results with regard to coping styles that might be important to dwell on. Active coping, for instance, was positively related to anxiety and PTSD symptoms among the Hungarian participants. According to Lazarus and Folkman (1984), people use different kinds of coping strategies depending on the nature of the stress and the efficacy of the same coping strategy, which may differ from one situation to another. Thus, how one perceives a situation (controllable vs. uncontrollable) may change the efficacy of the coping strategy (Lazarus and Folkman, 1984; Stanisławski, 2019). Our results suggest that active coping might have increased anxiety and PTSD symptoms among the Hungarian participants, because the situation they were in was not changing despite the efforts they made to change it. However, Lazarus and Folkman (1984) also stated that the efficacy of coping strategies may change in the long run. Thus, a coping strategy might be successful in the short term but then lose its efficacy in time, or the other way around (Sadaghiani and Sorkhab, 2013). Therefore, the active coping style might be dysfunctional temporarily for the Hungarian participants; however, it can develop into being functional in the long term. Future researchers may conduct longitudinal studies to investigate these possible associations. Another outcome was that emotional support was related to higher stress among the Norwegians, whereas it was related to lower anxiety among the Hungarians. One of the possible explanations for this discrepancy between the Norwegian and Hungarian participants can be that emotional support was positively correlated with approach acceptance among the Hungarian participants. However, emotional support was positively correlated with fear of death and negatively correlated with neutral acceptance among the Norwegian participants. Thus, these two death attitudes might mediate the relationship between emotional support and stress among the Norwegian participants, and having both the emotional support coping style and the approach acceptance death attitude may reduce anxiety among the Hungarian respondents. Another plausible explanation might be that the Norwegian participants in our study may not have gotten a right kind of social support when they needed it. Some researchers argued that sometimes an attempt to provide social support might result in higher psychological distress, as it can be experienced as intrusive, controlling, and directive by the recipient or the social support providers might give poor advice or fail to meet the certain needs of the person (Taylor, 2012). For example, if the person needs emotional support and, instead of this, gets only advice, then this can result in higher psychological distress (Taylor, 2012). Additionally, Sasaki and Yamasaki (2007) found that situational emotional support-seeking was related to higher somatic symptoms, anxiety, and insomnia. They explained that since emotional support-seeking does not focus on decreasing the stressor directly, the stressor can persist and may get even more powerful. Considering this explanation, our Norwegian participants might have used the emotional support coping style; however, as the pandemic continues to interrupt their daily lives, their stress levels might have continued to increase. However, similar to our previous explanation, this may change in the long run, and the emotional support coping style may become efficient in time. In addition, approach acceptance (believing in a happy afterlife) was negatively related to PTSD symptoms among the Hungarian participants. However, the religion coping style was related to higher PTSD symptoms in the Hungarian sample and higher anxiety in the Norwegian sample. In the literature, religiosity was divided into two dimensions (Neimeyer et al., 2004): (1) extrinsic religiosity, which displays a utilitarian perception of religion, and (2) intrinsic religiosity, in which people put faith at the center of their lives. Studies have demonstrated that intrinsic religiosity was related to lower death anxiety, whereas extrinsic religiosity was related to higher death anxiety (Neimeyer et al., 2004). Therefore, in our study, if the participants had mostly extrinsic religiosity, this may have increased their death anxiety, which may have led to higher levels of anxiety and PTSD symptoms. Lastly, the neutral acceptance death attitude was positively correlated with anxiety among the Turkish participants. For the Turkish participants, neutral acceptance was positively correlated with the acceptance coping style. However, this coping style was not associated with the mental health of our participants. Thus, it is possible that, for our Turkish sample, having only the neutral acceptance death attitude without some functional coping styles (e.g., positive reframing) might not be effective in buffering anxiety during COVID-19.

The analyses revealed some other results when we compared the relationships between the five death attitudes and the thirteen coping styles among the three countries that can be important to discuss. For instance, among the Norwegian and Turkish participants, informational support-seeking was positively correlated with fear of death, and for the Norwegian participants it was also positively associated with death avoidance. Some studies showed that obtaining information through social media was increasing anxiety among people during COVID-19, as the news mostly includes distressing and unreliable information (Xiong et al., 2020). Therefore, while seeking informational support, our Norwegian and Turkish participants might have been confronted with distressing and unreliable information, which might have resulted in higher fear of death and higher death avoidance for the Norwegian sample. In addition, emotional support was positively correlated with fear of death and negatively associated with neutral acceptance among the Norwegian respondents. Fino et al. (2020, 2021b) emphasized that physical and social isolation precautions related to COVID-19 might result in increased fear and distress regarding the disease itself among people. Additionally, some studies showed that stress may increase one’s likelihood of seeking emotional support (Joo et al., 2020). Therefore, in our Norwegian sample, the relationship between emotional support and fear of death and neutral acceptance may be the other way around; that is, the higher fear of death level of the participants might have triggered their involvement in emotional support, and people with higher neutral acceptance may not feel the need to engage in emotional support. Lastly, humor was positively associated with escape acceptance for both the Turkish and Norwegian participants. We may conclude that viewing death as an escape from a painful life was associated with use of the humor coping style among these participants.

Lastly, the analyses of this study for the control variables exhibited that the three countries also differ in terms of the relationship of age and gender with depression, anxiety, stress, and PTSD symptoms. For example, after the independent variables were entered into the regression in the final step, age was significantly and positively correlated with stress among the Norwegian participants, whereas it was significantly and negatively correlated with stress among the Turkish participants. On the other hand, age was not related to stress among the Hungarian participants. In addition, the Hungarian female participants reported significantly higher levels of anxiety than the Hungarian male participants in all the steps. However, gender was not a significant predictor among the Norwegian and Turkish participants in any of the steps. Previous studies have shown inconsistent results regarding the effect of gender on coronavirus anxiety (Spitzenstätter and Schnell, 2020). As discussed above, in our study, both the gender and age variables also show inconsistent outcomes among the three countries. Therefore, further studies are needed to examine the effect of these variables on mental health with regard to the pandemic.

### Limitations

While interpreting the results of this study, certain limitations should be taken into consideration. First, the method of convenience sampling was applied, as it has certain advantages (e.g., it is easy to carry out, relatively time efficient, and inexpensive). However, it also has drawbacks, such as inability to generalize the findings of the study to the population as a whole. For instance, in this research, the participants were psychology students. The results can, therefore, not be extrapolated to the entire population of Hungary, Norway, and Turkey. Moreover, the relatively small sample size used in this study might have increased the chance of committing a type II error (a false negative: rejecting statistically significant relationships when in fact there are). Therefore, one must be cautions while interpreting the results of this research. Thus, we may base the results of this study as exploratory. As a result, the significant findings may still be useful and give us some direction for future studies. Therefore, we encourage further research to replicate our study with larger sample size. Furthermore, the inconsistent results mentioned in the discussion part related to gender and age differences might be due to unbalanced samples in this study. Similarly, further studies with more balanced sample are needed to make these findings sounder. Lastly, as this study was correlational research, it is not possible to draw a causal interpretation from our outcomes.

Notwithstanding these limitations, this study was unique in terms of investigating different death attitudes related to COVID-19 among the three countries. In addition, by checking the effects on mental health and coping styles of the participants, we were able to see how the three variables (different death attitudes, coping styles, and mental health) might be related to each other and to COVID-19 among our sample in the three countries.

## Conclusion, Implications, and Suggestions for Future Studies

Our results suggested that having the escape acceptance death attitude and using some dysfunctional coping styles (self-blame, behavioral disengagement, self-distraction, and substance use) were related to lower mental health during COVID-19 in our sample. Practitioners in the field might pay closer attention to patients with higher escape acceptance and those who use dysfunctional coping styles. In addition, our findings implied that death attitudes and coping styles may differ in their efficacy among different countries. For instance, the fear of death attitude and behavioral disengagement coping style can be particularly risky for the Turkish participants. Therefore, practitioners in Turkey can pay more attention to this death attitude and to behavioral disengagement while working with patients during COVID-19. In addition, substance use might be particularly risky for the Hungarian participants. Similarly, practitioners in Hungary can pay more attention to this coping style while working with patients during COVID-19. Furthermore, positive reframing can be more functional among the Turkish and Hungarian participants, while humor might be more effective for the Norwegian participants. Therefore, practitioners may encourage their clients using techniques involving positive reframing in Turkey and Hungary, and in Norway they might provide techniques involving humor. Lastly, our analyses indicated that using active coping styles can be related to lower mental health in the short term, but that it might become functional in the long term among our Hungarian sample. Thus, practitioners should be cautious when working with clients with this coping style, and alert them of this possibility. We consider that the practical implications of this study can be useful in other similar contexts as well as in possible future outbreaks. Further studies can be conducted to investigate the relationship of mental health with death attitudes and coping styles, considering their long-term efficacy among different countries.

## Data Availability Statement

The raw data supporting the conclusions of this article will be made available by the authors, without undue reservation.

## Ethics Statement

The studies involving human participants were reviewed and approved and the ethical permission was obtained from the Institution of Review Board of ELTE Eötvös Loránd University (reference number: 2020/166). The patients/participants provided their written informed consent to participate in this study.

## Author Contributions

KO and MR worked on the statistical analysis. KO wrote the manuscript. MR and ÁS reviewed the manuscript and provided critical feedback. All authors made substantial contributions to conception, design, and data collection and interpretation of the data.

## Conflict of Interest

The authors declare that the research was conducted in the absence of any commercial or financial relationships that could be construed as a potential conflict of interest.

## Publisher’s Note

All claims expressed in this article are solely those of the authors and do not necessarily represent those of their affiliated organizations, or those of the publisher, the editors and the reviewers. Any product that may be evaluated in this article, or claim that may be made by its manufacturer, is not guaranteed or endorsed by the publisher.
